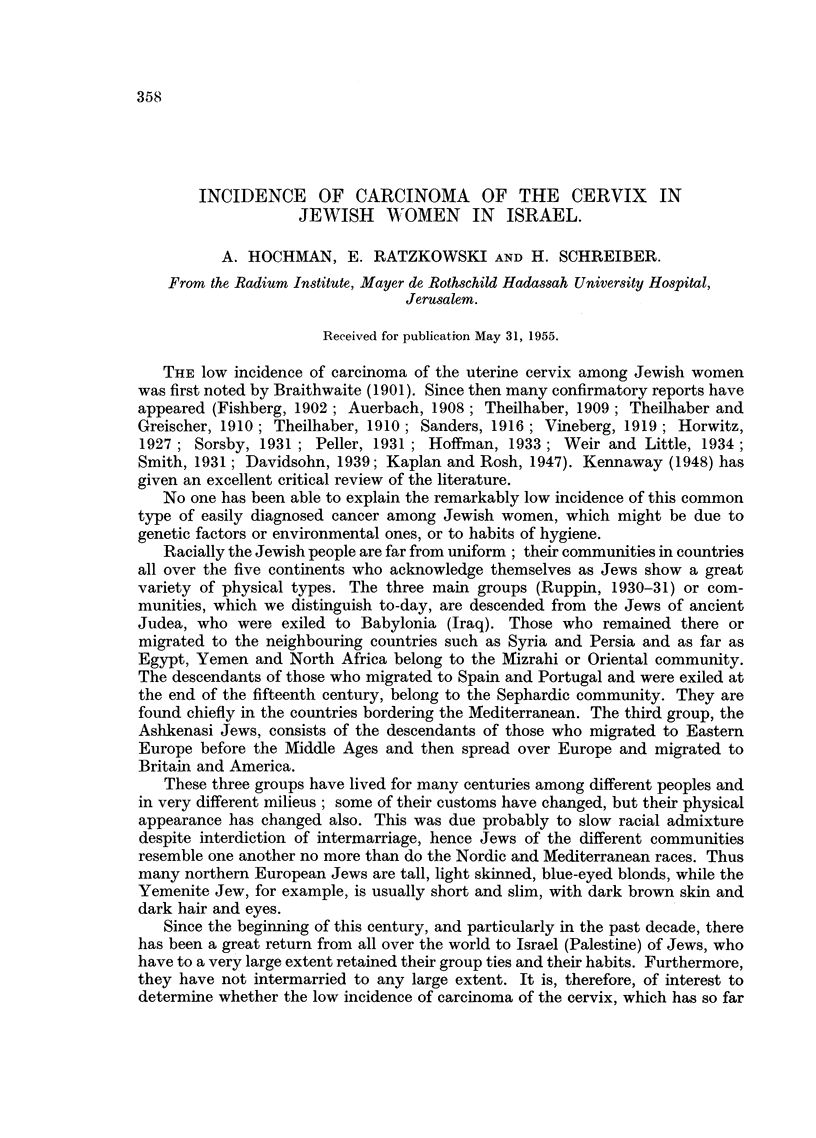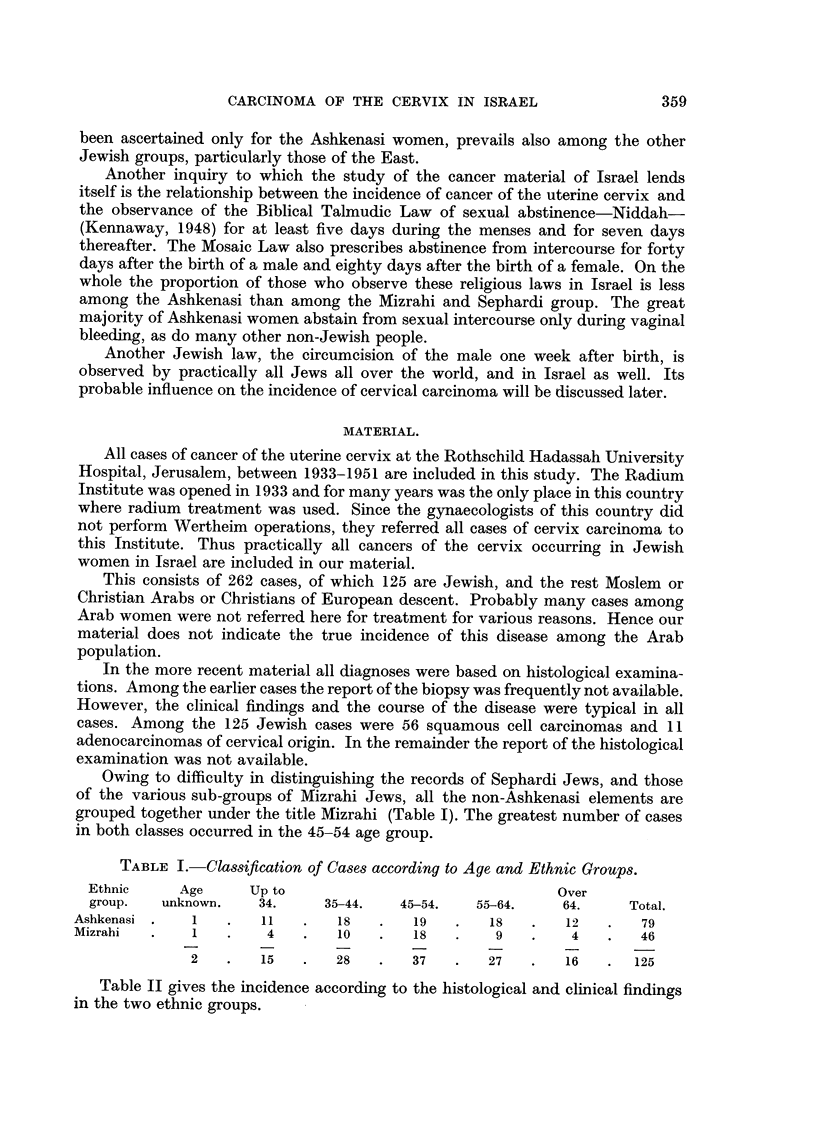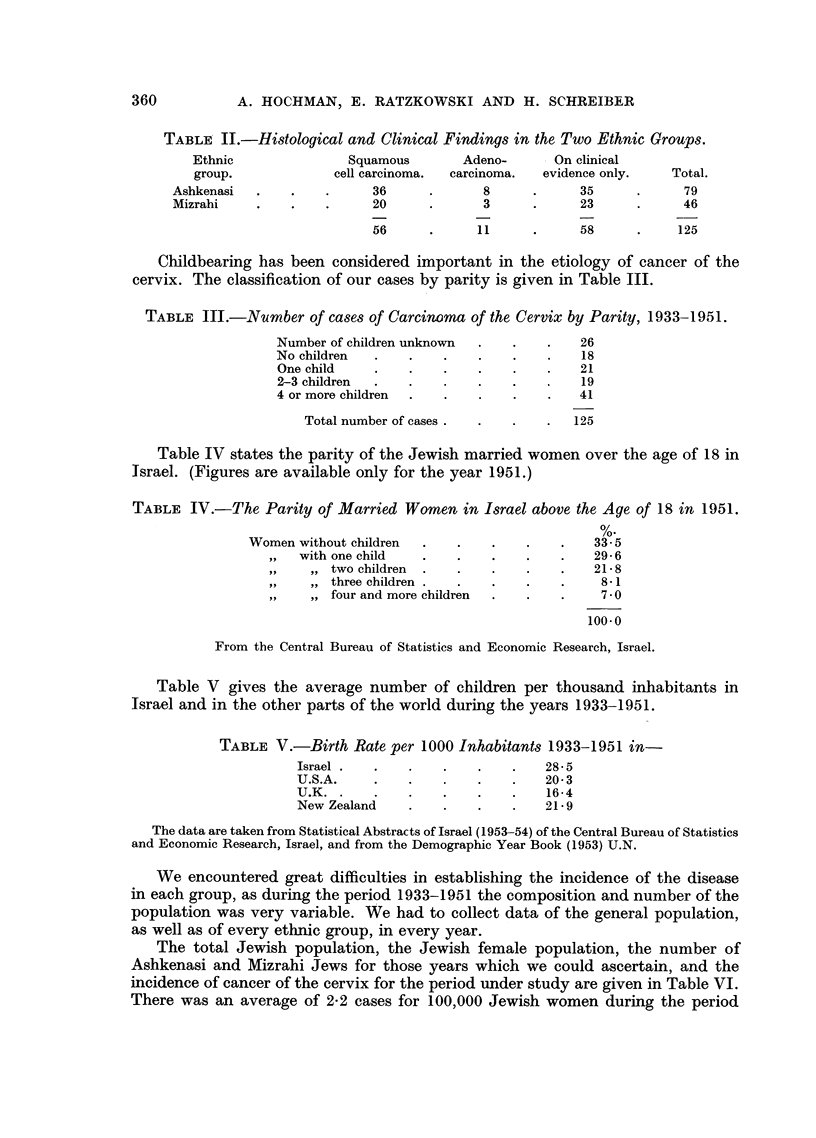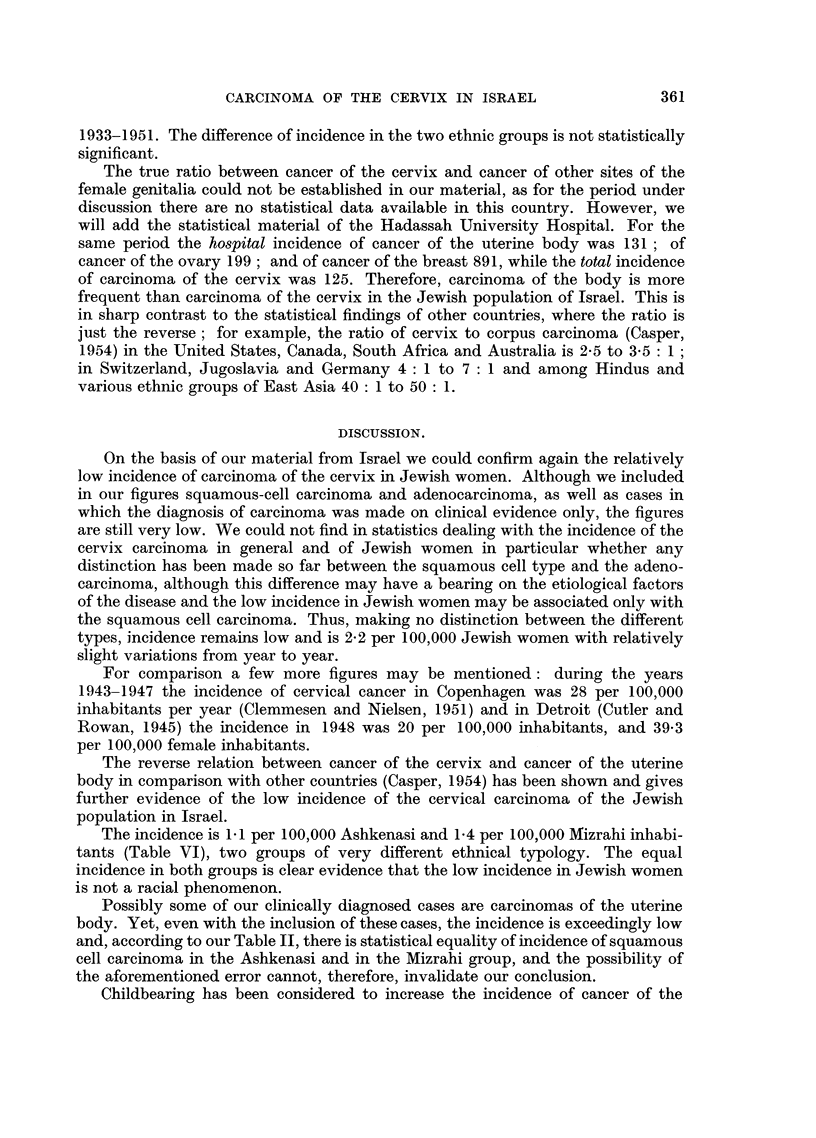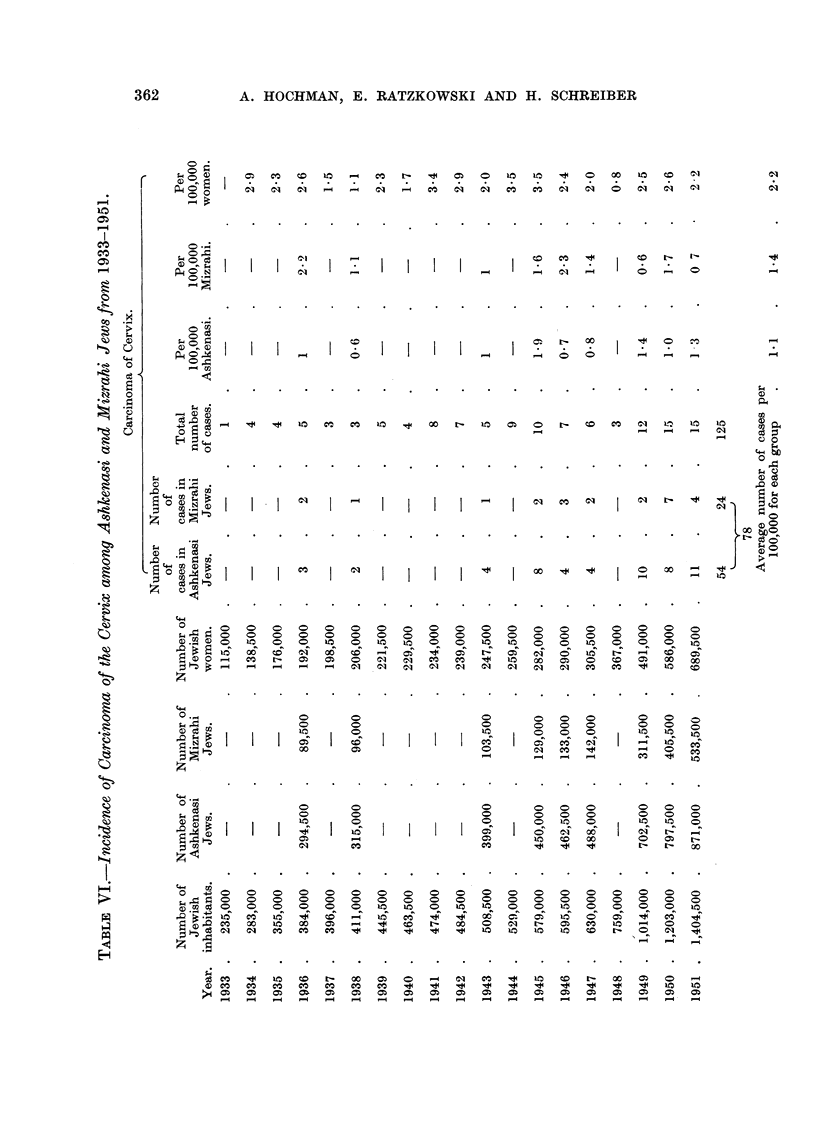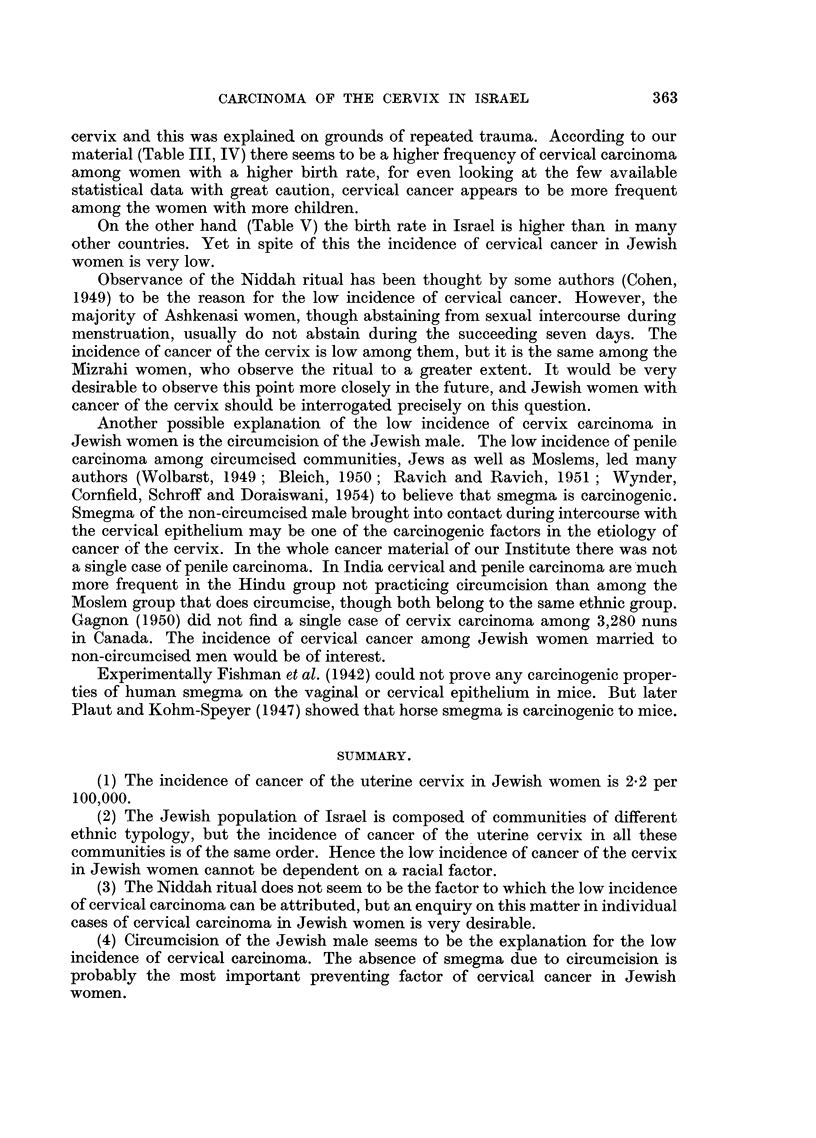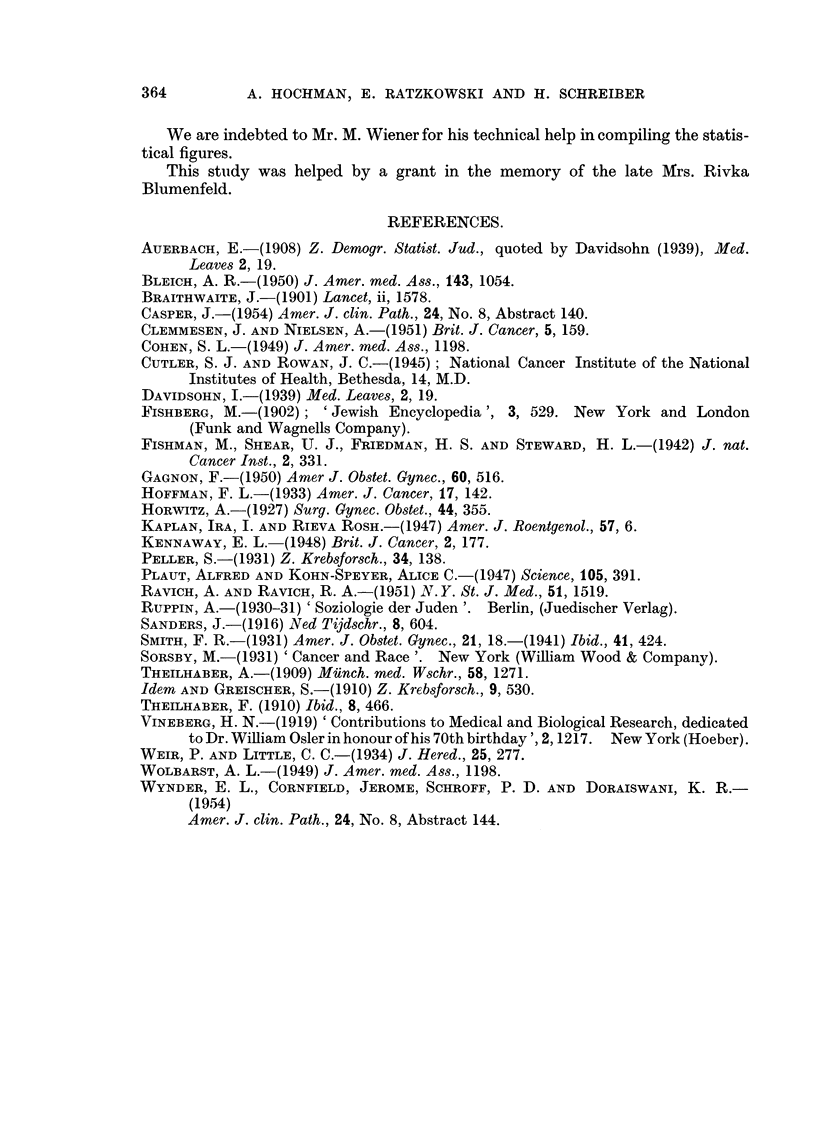# Incidence of Carcinoma of the Cervix in Jewish Women in Israel

**DOI:** 10.1038/bjc.1955.34

**Published:** 1955-09

**Authors:** A. Hochman, E. Ratzkowski, H. Schreiber


### INCIDENCE OF CARCINOMA OF THE CERVIX IN JEWISH WOMEN IN ISRAEL.

A. HOCHMAN, E. RATZKOWSKI AND H. SCHREIBER.

*From the Radium Institute, Mayer de Rothschild Hadassah University Hospital, Jerusalem.*

Received for publication May 31, 1955.

THE low incidence of carcinoma of the uterine cervix among Jewish women was first noted by Braithwaite (1901). Since then many confirmatory reports have appeared (Fishberg, 1902 ; Auerbach, 1908 ; Theilhaber, 1909 ; Theilhaber and Greischer, 1910 ; Theilhaber, 1910 ; Sanders, 1916 ; Vineberg, 1919 ; Horwitz, 1927 ; Sorsby, 1931 ; Peller, 1931 ; Hoffman, 1933 ; Weir and Little, 1934 ; Smith, 1931 ; Davidsohn, 1939 ; Kaplan and Rosh, 1947). Kennaway (1948) has given an excellent critical review of the literature.

No one has been able to explain the remarkably low incidence of this common type of easily diagnosed cancer among Jewish women, which might be due to genetic factors or environmental ones, or to habits of hygiene.

Racially the Jewish people are far from uniform ; their communities in countries all over the five continents who acknowledge themselves as Jews show a great variety of physical types. The three main groups (Ruppin, 1930–31) or communities, which we distinguish to-day, are descended from the Jews of ancient Judea, who were exiled to Babylonia (Iraq). Those who remained there or migrated to the neighbouring countries such as Syria and Persia and as far as Egypt, Yemen and North Africa belong to the Mizrahi or Oriental community. The descendants of those who migrated to Spain and Portugal and were exiled at the end of the fifteenth century, belong to the Sephardic community. They are found chiefly in the countries bordering the Mediterranean. The third group, the Ashkenasi Jews, consists of the descendants of those who migrated to Eastern Europe before the Middle Ages and then spread over Europe and migrated to Britain and America.

These three groups have lived for many centuries among different peoples and in very different milieus ; some of their customs have changed, but their physical appearance has changed also. This was due probably to slow racial admixture despite interdiction of intermarriage, hence Jews of the different communities resemble one another no more than do the Nordic and Mediterranean races. Thus many northern European Jews are tall, light skinned, blue-eyed blonds, while the Yemenite Jew, for example, is usually short and slim, with dark brown skin and dark hair and eyes.

Since the beginning of this century, and particularly in the past decade, there has been a great return from all over the world to Israel (Palestine) of Jews, who have to a very large extent retained their group ties and their habits. Furthermore, they have not intermarried to any large extent. It is, therefore, of interest to determine whether the low incidence of carcinoma of the cervix, which has so far been ascertained only for the Ashkenasi women, prevails also among the other Jewish groups, particularly those of the East.

Another inquiry to which the study of the cancer material of Israel lends itself is the relationship between the incidence of cancer of the uterine cervix and the observance of the Biblical Talmudic Law of sexual abstinence—Niddah—(Kennaway, 1948) for at least five days during the menses and for seven days thereafter. The Mosaic Law also prescribes abstinence from intercourse for forty days after the birth of a male and eighty days after the birth of a female. On the whole the proportion of those who observe these religious laws in Israel is less among the Ashkenasi than among the Mizrahi and Sephardi group. The great majority of Ashkenasi women abstain from sexual intercourse only during vaginal bleeding, as do many other non-Jewish people.

Another Jewish law, the circumcision of the male one week after birth, is observed by practically all Jews all over the world, and in Israel as well. Its probable influence on the incidence of cervical carcinoma will be discussed later.

### MATERIAL.

All cases of cancer of the uterine cervix at the Rothschild Hadassah University Hospital, Jerusalem, between 1933–1951 are included in this study. The Radium Institute was opened in 1933 and for many years was the only place in this country where radium treatment was used. Since the gynaecologists of this country did not perform Wertheim operations, they referred all cases of cervix carcinoma to this Institute. Thus practically all cancers of the cervix occurring in Jewish women in Israel are included in our material.

This consists of 262 cases, of which 125 are Jewish, and the rest Moslem or Christian Arabs or Christians of European descent. Probably many cases among Arab women were not referred here for treatment for various reasons. Hence our material does not indicate the true incidence of this disease among the Arab population.

In the more recent material all diagnoses were based on histological examinations. Among the earlier cases the report of the biopsy was frequently not available. However, the clinical findings and the course of the disease were typical in all cases. Among the 125 Jewish cases were 56 squamous cell carcinomas and 11 adenocarcinomas of cervical origin. In the remainder the report of the histological examination was not available.

Owing to difficulty in distinguishing the records of Sephardi Jews, and those of the various sub-groups of Mizrahi Jews, all the non-Ashkenasi elements are grouped together under the title Mizrahi (Table I). The greatest number of cases in both classes occurred in the 45–54 age group.

TABLE I.—*Classification of Cases according to Age and Ethnic Groups.*

| Ethnic group. | Age unknown. | Up to 34. | 35–44. | 45–54. | 55–64. | Over 64. | Total. |
|---|---|---|---|---|---|---|---|
| Ashkenasi | 1 | 11 | 18 | 19 | 18 | 12 | 79 |
| Mizrahi | 1 | 4 | 10 | 18 | 9 | 4 | 46 |
| | 2 | 15 | 28 | 37 | 27 | 16 | 125 |

Table II gives the incidence according to the histological and clinical findings in the two ethnic groups.

TABLE II.—*Histological and Clinical Findings in the Two Ethnic Groups.*

| Ethnic group. | Squamous cell carcinoma. | Adeno-carcinoma. | On clinical evidence only. | Total. |
|---|---|---|---|---|
| Ashkenasi | 36 | 8 | 35 | 79 |
| Mizrahi | 20 | 3 | 23 | 46 |
| | 56 | 11 | 58 | 125 |

Childbearing has been considered important in the etiology of cancer of the cervix. The classification of our cases by parity is given in Table III.

TABLE III.—*Number of cases of Carcinoma of the Cervix by Parity,* 1933–1951.

| | |
|---|---|
| Number of children unknown | 26 |
| No children | 18 |
| One child | 21 |
| 2–3 children | 19 |
| 4 or more children | 41 |
| Total number of cases | 125 |

Table IV states the parity of the Jewish married women over the age of 18 in Israel. (Figures are available only for the year 1951.)

TABLE IV.—*The Parity of Married Women in Israel above the Age of* 18 *in* 1951.

| | %. |
|---|---|
| Women without children | 33·5 |
| ,, with one child | 29·6 |
| ,, ,, two children | 21·8 |
| ,, ,, three children | 8·1 |
| ,, ,, four and more children | 7·0 |
| | 100·0 |

From the Central Bureau of Statistics and Economic Research, Israel.

Table V gives the average number of children per thousand inhabitants in Israel and in the other parts of the world during the years 1933–1951.

TABLE V.—*Birth Rate per* 1000 *Inhabitants* 1933–1951 *in*—

| | |
|---|---|
| Israel | 28·5 |
| U.S.A. | 20·3 |
| U.K. | 16·4 |
| New Zealand | 21·9 |

The data are taken from Statistical Abstracts of Israel (1953–54) of the Central Bureau of Statistics and Economic Research, Israel, and from the Demographic Year Book (1953) U.N.

We encountered great difficulties in establishing the incidence of the disease in each group, as during the period 1933–1951 the composition and number of the population was very variable. We had to collect data of the general population, as well as of every ethnic group, in every year.

The total Jewish population, the Jewish female population, the number of Ashkenasi and Mizrahi Jews for those years which we could ascertain, and the incidence of cancer of the cervix for the period under study are given in Table VI. There was an average of 2·2 cases for 100,000 Jewish women during the period

1933–1951. The difference of incidence in the two ethnic groups is not statistically significant.

The true ratio between cancer of the cervix and cancer of other sites of the female genitalia could not be established in our material, as for the period under discussion there are no statistical data available in this country. However, we will add the statistical material of the Hadassah University Hospital. For the same period the *hospital* incidence of cancer of the uterine body was 131 ; of cancer of the ovary 199 ; and of cancer of the breast 891, while the *total* incidence of carcinoma of the cervix was 125. Therefore, carcinoma of the body is more frequent than carcinoma of the cervix in the Jewish population of Israel. This is in sharp contrast to the statistical findings of other countries, where the ratio is just the reverse ; for example, the ratio of cervix to corpus carcinoma (Casper, 1954) in the United States, Canada, South Africa and Australia is 2·5 to 3·5 : 1 ; in Switzerland, Jugoslavia and Germany 4 : 1 to 7 : 1 and among Hindus and various ethnic groups of East Asia 40 : 1 to 50 : 1.

### DISCUSSION.

On the basis of our material from Israel we could confirm again the relatively low incidence of carcinoma of the cervix in Jewish women. Although we included in our figures squamous-cell carcinoma and adenocarcinoma, as well as cases in which the diagnosis of carcinoma was made on clinical evidence only, the figures are still very low. We could not find in statistics dealing with the incidence of the cervix carcinoma in general and of Jewish women in particular whether any distinction has been made so far between the squamous cell type and the adenocarcinoma, although this difference may have a bearing on the etiological factors of the disease and the low incidence in Jewish women may be associated only with the squamous cell carcinoma. Thus, making no distinction between the different types, incidence remains low and is 2·2 per 100,000 Jewish women with relatively slight variations from year to year.

For comparison a few more figures may be mentioned : during the years 1943–1947 the incidence of cervical cancer in Copenhagen was 28 per 100,000 inhabitants per year (Clemmesen and Nielsen, 1951) and in Detroit (Cutler and Rowan, 1945) the incidence in 1948 was 20 per 100,000 inhabitants, and 39·3 per 100,000 female inhabitants.

The reverse relation between cancer of the cervix and cancer of the uterine body in comparison with other countries (Casper, 1954) has been shown and gives further evidence of the low incidence of the cervical carcinoma of the Jewish population in Israel.

The incidence is 1·1 per 100,000 Ashkenasi and 1·4 per 100,000 Mizrahi inhabitants (Table VI), two groups of very different ethnical typology. The equal incidence in both groups is clear evidence that the low incidence in Jewish women is not a racial phenomenon.

Possibly some of our clinically diagnosed cases are carcinomas of the uterine body. Yet, even with the inclusion of these cases, the incidence is exceedingly low and, according to our Table II, there is statistical equality of incidence of squamous cell carcinoma in the Ashkenasi and in the Mizrahi group, and the possibility of the aforementioned error cannot, therefore, invalidate our conclusion.

Childbearing has been considered to increase the incidence of cancer of the

TABLE VI.—*Incidence of Carcinoma of the Cervix among Ashkenasi and Mizrahi Jews from* 1933–1951.

| Year. | Number of Jewish inhabitants. | Number of Ashkenasi Jews. | Number of Mizrahi Jews. | Number of Jewish women. | Carcinoma of Cervix. Number of cases in Ashkenasi Jews. | Number of cases in Mizrahi Jews. | Total number of cases. | Per 100,000 Ashkenasi. | Per 100,000 Mizrahi. | Per 100,000 women. |
|---|---|---|---|---|---|---|---|---|---|---|
| 1933 | 235,000 | — | — | 115,000 | — | — | 1 | — | — | — |
| 1934 | 283,000 | — | — | 138,500 | — | — | 4 | — | — | 2·9 |
| 1935 | 355,000 | — | — | 176,000 | — | — | 4 | — | — | 2·3 |
| 1936 | 384,000 | 294,500 | 89,500 | 192,000 | 3 | 2 | 5 | 1 | 2·2 | 2·6 |
| 1937 | 396,000 | — | — | 198,500 | — | — | 3 | — | — | 1·5 |
| 1938 | 411,000 | 315,000 | 96,000 | 206,000 | 2 | 1 | 3 | 0·6 | 1·1 | 1·1 |
| 1939 | 445,500 | — | — | 221,500 | — | — | 5 | — | — | 2·3 |
| 1940 | 463,500 | — | — | 229,500 | — | — | 4 | — | — | 1·7 |
| 1941 | 474,000 | — | — | 234,000 | — | — | 8 | — | — | 3·4 |
| 1942 | 484,500 | — | — | 239,000 | — | — | 7 | — | — | 2·9 |
| 1943 | 508,500 | 399,000 | 103,500 | 247,500 | 4 | 1 | 5 | 1 | 1 | 2·0 |
| 1944 | 529,000 | — | — | 259,500 | — | — | 9 | — | — | 3·5 |
| 1945 | 579,000 | 450,000 | 129,000 | 282,000 | 8 | 2 | 10 | 1·9 | 1·6 | 3·5 |
| 1946 | 595,500 | 462,500 | 133,000 | 290,000 | 4 | 3 | 7 | 0·7 | 2·3 | 2·4 |
| 1947 | 630,000 | 488,000 | 142,000 | 305,500 | 4 | 2 | 6 | 0·8 | 1·4 | 2·0 |
| 1948 | 759,000 | — | — | 367,000 | — | — | 3 | — | — | 0·8 |
| 1949 | 1,014,000 | 702,500 | 311,500 | 491,000 | 10 | 2 | 12 | 1·4 | 0·6 | 2·5 |
| 1950 | 1,203,000 | 797,500 | 405,500 | 586,000 | 8 | 7 | 15 | 1·0 | 1·7 | 2·6 |
| 1951 | 1,404,500 | 871,000 | 533,500 | 689,500 | 11 | 4 | 15 | 1·3 | 0·7 | 2·2 |
| | | | | | 54 | 24 | 125 | | | |
| | | | | | 78 (54 + 24) | | | | | |
| | | | | | Average number of cases per 100,000 for each group | | | 1·1 | 1·4 | 2·2 |

cervix and this was explained on grounds of repeated trauma. According to our material (Table III, IV) there seems to be a higher frequency of cervical carcinoma among women with a higher birth rate, for even looking at the few available statistical data with great caution, cervical cancer appears to be more frequent among the women with more children.

On the other hand (Table V) the birth rate in Israel is higher than in many other countries. Yet in spite of this the incidence of cervical cancer in Jewish women is very low.

Observance of the Niddah ritual has been thought by some authors (Cohen, 1949) to be the reason for the low incidence of cervical cancer. However, the majority of Ashkenasi women, though abstaining from sexual intercourse during menstruation, usually do not abstain during the succeeding seven days. The incidence of cancer of the cervix is low among them, but it is the same among the Mizrahi women, who observe the ritual to a greater extent. It would be very desirable to observe this point more closely in the future, and Jewish women with cancer of the cervix should be interrogated precisely on this question.

Another possible explanation of the low incidence of cervix carcinoma in Jewish women is the circumcision of the Jewish male. The low incidence of penile carcinoma among circumcised communities, Jews as well as Moslems, led many authors (Wolbarst, 1949 ; Bleich, 1950 ; Ravich and Ravich, 1951 ; Wynder, Cornfield, Schroff and Doraiswani, 1954) to believe that smegma is carcinogenic. Smegma of the non-circumcised male brought into contact during intercourse with the cervical epithelium may be one of the carcinogenic factors in the etiology of cancer of the cervix. In the whole cancer material of our Institute there was not a single case of penile carcinoma. In India cervical and penile carcinoma are much more frequent in the Hindu group not practicing circumcision than among the Moslem group that does circumcise, though both belong to the same ethnic group. Gagnon (1950) did not find a single case of cervix carcinoma among 3,280 nuns in Canada. The incidence of cervical cancer among Jewish women married to non-circumcised men would be of interest.

Experimentally Fishman *et al.* (1942) could not prove any carcinogenic properties of human smegma on the vaginal or cervical epithelium in mice. But later Plaut and Kohm-Speyer (1947) showed that horse smegma is carcinogenic to mice.

### SUMMARY.

(1) The incidence of cancer of the uterine cervix in Jewish women is 2·2 per 100,000.

(2) The Jewish population of Israel is composed of communities of different ethnic typology, but the incidence of cancer of the uterine cervix in all these communities is of the same order. Hence the low incidence of cancer of the cervix in Jewish women cannot be dependent on a racial factor.

(3) The Niddah ritual does not seem to be the factor to which the low incidence of cervical carcinoma can be attributed, but an enquiry on this matter in individual cases of cervical carcinoma in Jewish women is very desirable.

(4) Circumcision of the Jewish male seems to be the explanation for the low incidence of cervical carcinoma. The absence of smegma due to circumcision is probably the most important preventing factor of cervical cancer in Jewish women.

We are indebted to Mr. M. Wiener for his technical help in compiling the statistical figures.

This study was helped by a grant in the memory of the late Mrs. Rivka Blumenfeld.

### REFERENCES.

AUERBACH, E.—(1908) *Z. Demogr. Statist. Jud.*, quoted by Davidsohn (1939), *Med. Leaves* **2**, 19.

BLEICH, A. R.—(1950) *J. Amer. med. Ass.*, **143**, 1054.

BRAITHWAITE, J.—(1901) *Lancet*, ii, 1578.

CASPER, J.—(1954) *Amer. J. clin. Path.*, **24**, No. 8, Abstract 140.

CLEMMESEN, J. AND NIELSEN, A.—(1951) *Brit. J. Cancer*, **5**, 159.

COHEN, S. L.—(1949) *J. Amer. med. Ass.*, 1198.

CUTLER, S. J. AND ROWAN, J. C.—(1945) ; National Cancer Institute of the National Institutes of Health, Bethesda, 14, M.D.

DAVIDSOHN, I.—(1939) *Med. Leaves*, **2**, 19.

FISHBERG, M.—(1902) ; 'Jewish Encyclopedia', **3**, 529. New York and London (Funk and Wagnells Company).

FISHMAN, M., SHEAR, U. J., FRIEDMAN, H. S. AND STEWARD, H. L.—(1942) *J. nat. Cancer Inst.*, **2**, 331.

GAGNON, F.—(1950) *Amer J. Obstet. Gynec.*, **60**, 516.

HOFFMAN, F. L.—(1933) *Amer. J. Cancer*, **17**, 142.

HORWITZ, A.—(1927) *Surg. Gynec. Obstet.*, **44**, 355.

KAPLAN, IRA, I. AND RIEVA ROSH.—(1947) *Amer. J. Roentgenol.*, **57**, 6.

KENNAWAY, E. L.—(1948) *Brit. J. Cancer*, **2**, 177.

PELLER, S.—(1931) *Z. Krebsforsch.*, **34**, 138.

PLAUT, ALFRED AND KOHN-SPEYER, ALICE C.—(1947) *Science*, **105**, 391.

RAVICH, A. AND RAVICH, R. A.—(1951) *N.Y. St. J. Med.*, **51**, 1519.

RUPPIN, A.—(1930–31) 'Soziologie der Juden'. Berlin, (Juedischer Verlag).

SANDERS, J.—(1916) *Ned Tijdschr.*, **8**, 604.

SMITH, F. R.—(1931) *Amer. J. Obstet. Gynec.*, **21**, 18.—(1941) *Ibid.*, **41**, 424.

SORSBY, M.—(1931) 'Cancer and Race'. New York (William Wood & Company).

THEILHABER, A.—(1909) *Münch. med. Wschr.*, **58**, 1271.

*Idem* AND GREISCHER, S.—(1910) *Z. Krebsforsch.*, **9**, 530.

THEILHABER, F. (1910) *Ibid.*, **8**, 466.

VINEBERG, H. N.—(1919) 'Contributions to Medical and Biological Research, dedicated to Dr. William Osler in honour of his 70th birthday', **2**, 1217. New York (Hoeber).

WEIR, P. AND LITTLE, C. C.—(1934) *J. Hered.*, **25**, 277.

WOLBARST, A. L.—(1949) *J. Amer. med. Ass.*, 1198.

WYNDER, E. L., CORNFIELD, JEROME, SCHROFF, P. D. AND DORAISWANI, K. R.—(1954)
*Amer. J. clin. Path.*, **24**, No. 8, Abstract 144.